# Adverse outcomes following psychedelic use in adolescents and adults: associations with age and personality traits

**DOI:** 10.1186/s13034-026-01048-x

**Published:** 2026-02-24

**Authors:** David Sjöström, Emma Claesdotter-Knutsson, Petri Kajonius

**Affiliations:** 1 Department of Clinical Sciences, Faculty of Medicine, Lund University, Lund, Sweden; 2 Region Skåne, Child and Adolescent Psychiatry, Skane University Hospital, Lund, Sweden; 3 Department of Psychology, Lund University, Lund, Sweden

**Keywords:** Adolescents, Psychedelics, Adverse outcomes, Personality traits

## Abstract

**Background:**

Adolescents are increasingly using classical psychedelics, yet little is known about how psychedelics use is related to their mental health. Emerging evidence suggests that adolescents may be more vulnerable to adverse outcomes in relation to psychedelic use compared to adults. This descriptive and exploratory study examined differences between adolescents and adults in reported psychedelic experiences, with a focus on adverse outcomes and the potential role of personality traits.

**Methods:**

Data were drawn from a community sample (*N* = 1185), in which participants retrospectively reported on their most significant psychedelic experience and associations with adverse (e.g., confusion) and positive outcomes (e.g., meaningfulness). The sample was divided into adolescents aged 18–24 years and adults aged 25 years or older. Age groups were analysed both dichotomously (< 25 vs. ≥25 years) and continuously. Analyses of covariance (ANCOVA) and linear regressions were used to test the role of age and personality traits as predictors of adverse outcomes.

**Results:**

Adolescents reported significantly more adverse outcomes compared to adults, including more negative personality change as well as more fearful experiences. Positive outcomes such as meaningfulness, mystical-type experiences, and improvements in relationships did not differ significantly between age groups. Age group remained a significant predictor of adverse outcomes after adjusting for personality traits. Neuroticism explained a substantially larger share of variance compared to age.

**Conclusions:**

These findings suggest that while adolescents may derive similar positive effects from psychedelics as adults, they may be more vulnerable to adverse outcomes. The findings underscore the need for further longitudinal research to understand how developmental stages and individual differences influence psychedelic use outcomes.

**Supplementary Information:**

The online version contains supplementary material available at 10.1186/s13034-026-01048-x.

## Introduction

Initial reports suggest a differential pattern in adolescents’ responses to psychedelic use compared to adults. While both age groups may report improvements in psychological well-being, adolescents appear more prone to challenging experiences and post-experience adverse outcomes [1–3]. Considering the increasing prevalence of psychedelic use among adolescents [4–6], these potential age-related differences warrant further systematic investigation to inform evidence-based harm-reduction strategies and future research.

Adolescence in the present study is defined as a developmental period in life spanning ages 10 to 24 [7], although researchers also define the age of 18–25 as emerging adulthood depending on the theoretical framework [8]. In legal and clinical trial contexts the age of 18 is commonly used as a practical cutoff between adolescents (minors) and adults, although adolescent development changes in brain structure, emotional regulation, identity formation, and decision-making capacity are still in process after the age of 18 with individual variations [9]. A developmental mismatch is often observed between the earlier maturation of the limbic system, which regulates emotion and reward, and the later maturation of the prefrontal cortex, which supports executive control and risk assessment [10]. Recent evolutionary–developmental perspectives further emphasize that the adolescent brain is uniquely adapted for exploration, reward sensitivity, and social learning. While these features facilitate adaptive development, they may also heighten vulnerability to risk-taking and maladaptive behaviours [11]. How these hallmarks of adolescence manifest in relation to use of psychoactive drugs such as classical psychedelics and adverse outcomes is mostly unknown [4].

The interest in naturalistic use of classical psychedelics—such as LSD, psilocybin, and ayahuasca—has resurged in recent years [12]. At sufficient doses, psychedelics reliably induce altered states of consciousness characterized by profound changes in cognition, perception, and emotion [13]. Initiation most often occurs during late adolescence, with prevalence of use highest between ages 20–29 compared to any other stage of life [14]. In Sweden, the 2024 National School Survey found that 1.9% of students in grades 9 and 11 reported lifetime psychedelic use [15], and data from the Swedish Twin Registry indicated a lifetime prevalence of 3–4% among the same age group [16].

Age-group-related effects have been proposed in cannabis use during adolescence, with more risk of psychiatric disorders and cognitive impairment compared to adult-onset use, and different acute effects of cannabis compared to adults, although findings are currently mixed and related to individual differences [17–20]. Adolescents also appear to respond differently to prescribed psychopharmacological treatments. SSRIs and antipsychotics often show reduced efficacy in the adolescent age group compared to adults [21, 22], whereas ADHD medications tend to produce more favourable responses in adolescents relative to adults, although more research is needed in regards to individual differences and responses [23]. These findings again highlight the importance of age-specific research on psychedelic effects on mental health and adding individual differences as covariates.

In adults (in most studies defined as above age 18 or 21), both clinical trials and naturalistic studies demonstrate that classical psychedelics can lead to sustained improvements in mental health outcomes such as wellbeing, including reductions in depression, anxiety, PTSD, eating disorders, and substance use disorders [12, 24, 25] and with low risk for mental and physical harm [26, 27]. Psychedelic experiences have been reported to influence interpersonal connections and nature relatedness, including increased perceptions of closeness, empathy, and social connectedness, making relational outcomes a relevant exploratory dimension alongside intrapersonal effects [28, 29]. In contrast, evidence specific to how adolescents experience mental health effects of classical psychedelics remains very limited and is called for in the literature [4, 30, 31]. A prospective naturalistic study comparing adolescents (age group 16–24 years) and adults (age group 25+) found that both age groups reported improvements in psychological well-being following psychedelic use; however, adolescents more frequently endorsed challenging psychedelic experiences and perceptual side effects [3]. A mix-methods naturalistic study of ayahuasca use among adolescents in religious contexts in Brazil reported improved mental health indicators and no significant neuropsychological deficits compared to age matched controls [32, 33]. These initial studies, despite important limitations, suggest that adolescents may as well as adults report benefits with psychedelic use, although indicating more sensitivity to the acute psychological effects.

Personality psychology seeks to describe and explain the enduring patterns of thinking, feeling, and behaving that make individuals unique [34]. Personality traits can further be defined as basic tendencies of individual differences that are stable across time and consistent across situations. Within this framework, the Five-Factor Model—commonly referred to as the Big Five—has emerged as a prominent universal taxonomy for personality structure [35]. The model identifies five broad domains—neuroticism (tendency toward negative emotionality and stress reactivity), extraversion (sociability, assertiveness, and energetic engagement), openness to experience (intellectual curiosity, imagination, and aesthetic sensitivity), agreeableness (compassion, cooperation, and trust), and conscientiousness (organization, diligence, and self-control). Extensive cross-cultural evidence supports the view that these five dimensions capture the most fundamental aspects of human personality variation, making the Big Five the dominant paradigm for studying individual differences in psychology and their relationships to mental health [36].

In research on psychedelics including personality traits, neuroticism has consistently been linked to a higher likelihood of challenging psychedelic experiences, whereas openness and absorption have been associated with beneficial outcomes, including an increased probability of mystical-type experiences [37, 38]. Importantly, adolescents generally score higher on neuroticism compared to adults [39], a developmental stage related difference that may explain part of the age group differences in psychedelic responses. Recent studies comparing psychedelic users and non-users further indicate that controlling for neuroticism significantly increases the explained variance in mental health outcomes [15, 40], underscoring the relevance of including personality as an exploratory factor in prediction models.

In summary, this descriptive and exploratory study pursued three research objectives. First, we sought to compare adolescents (< 25) and adults (≥ 25) with respect to adverse outcomes related to psychedelic use, hypothesising that adolescents would report more adverse outcomes. Second, we aimed at to test to what extent individual personality traits account for variance in adverse outcomes, hypothesising that neuroticism would be more strongly associated. Third, we investigated how age used as a continuous variable contrasted with dichotomous grouping (< 25 vs. ≥25) in predicting adverse outcomes, hypothesising that age effects would be different depending on the model and less when age is included continuously.

## Method

### Procedure and sample

Cross-sectional data were collected between October and December 2024 via an online survey targeting individuals with prior experience of psychedelic substances. The survey was designed by the research team using Google Forms. Participants were recruited through direct contacts, social media postings, and newsletters distributed to communities with an interest in psychedelic use. In addition, a second recruitment wave was conducted through *Prolific* (prolific.com), an established online platform widely used for behavioural and psychological research. Prolific offers access to a diverse participant pool and allows pre-screening based on demographic and psychological variables. Data from the Prolific subsample were collected to address a separate hypothesis in a different analysis. These analyses are reported in a separate manuscript currently under review. For Prolific recruitment, we used the platform’s built-in prescreening tools, including demographic filters and the option to approximate population distributions on selected age and gender characteristics. It is important to note that Prolific recruitment does not yield a statistically representative sample of the general population. Accordingly, no claims of population representativeness are made. However, several peer-reviewed studies have demonstrated that Prolific samples generally show high data quality relative to other online recruitment platforms, including lower rates of inattentive responding, higher compliance with instructions, and more reliable psychometric properties [41]. Participants recruited via Prolific received standard monetary compensation in accordance with Prolific’s platform guidelines, whereas participants recruited through social media did not receive financial incentives.

Several steps were taken to enhance data validity and reduce the likelihood of non-human or inattentive responding. First, the survey was administered using Google Forms with CAPTCHA verification enabled, which served as a basic safeguard against automated bot submissions. Second, all responses were visually inspected prior to analysis to identify implausible response patterns (e.g., uniform responding across scales, inconsistent demographic information, or clearly erratic entries). No systematic patterns indicative of automated or invalid responding was identified.

In addition, internal validity was indirectly evaluated through examination of theoretically expected associations among key variables. For example, neuroticism showed expected associations with lower well-being and more adverse outcomes, consistent with prior literature, and differed by sex in directions previously reported. These convergent patterns provide indirect support for the plausibility of the data and the likelihood that responses reflect meaningful individual differences rather than random or automated input.

The final analytic sample consisted of 1,185 individuals with self-reported prior use of a classic psychedelic substance. Participants were categorized into two age groups: adolescents (< 25 years; *N* = 134) and adults (≥ 25 years; *N* = 1,051). In the adolescent group, 62.8% identified as male, 34.9% as female, and 1.3% as “other.” In the adult group, 58.3% identified as male, 40.9% as female, and 0.1% as “other.” Missing data were minimal (three cases across the total sample).

Inclusion criteria required participants to be at least 16 years of age, to have prior experience with a classic psychedelic substance, and to be able to complete the online survey in either Swedish or English. Informed consent was obtained electronically before participation. No exclusion criteria were applied.

### Instruments

#### Wellbeing

Subjective wellbeing was assessed using The Harmony in Life Scale (HILS), a validated 3-item scale measuring perceived harmony and balance in life [42]. Participants responded using a 7-point Likert scale ranging from 1 (strongly disagree) to 7 (strongly agree). Internal consistency was acceptable (Cronbach’s α = 0.89). The Life Satisfaction Index (LSI-4) was computed as the mean of four items assessing satisfaction with health, family relationships, friendships, and life overall, with each item rated on a 5-point scale from 1 (very dissatisfied) to 5 (very satisfied). Health satisfaction was assessed using a single-item general health rating, also on a 5-point scale ranging from 1 (very poor) to 5 (very good).

#### Personality

The validated Big Five IPIP-NEO-30 was used to assess the five major personality traits: Neuroticism, Extraversion, Openness, Agreeableness, and Conscientiousness [35]. The inventory comprised 30 items rated on a 5-point Likert scale ranging from 1 (strongly disagree) to 5 (strongly agree). Composite scores for each personality trait were computed by averaging the relevant items. Internal consistency was acceptable across all domains (Cronbach’s α = 0.90 for Neuroticism, α = 0.80 for Extraversion, α = 0.80 for Openness, α = 0.70 for Agreeableness, and α = 0.80 for Conscientiousness).

#### Adverse outcome index

To evaluate the perceived impact of participants’ most significant psychedelic experience on mental health, a 7-item Adverse Outcome Index was constructed by the research team including common side-effects in relation to psychedelic use. This index assessed the presence and severity of post-experience difficulties, including confusion, persistent anxiety, mood disturbance, sensory disturbances, sleep problems, hyperactivation, and feelings of unreality. Items were rated on a 5-point Likert scale ranging from 1 (not at all) to 5 (very much). Composite scores were computed as the mean of all items (Cronbach’s α = 0.90).

#### Personality change

Participants were asked to evaluate whether they experienced lasting changes in their personality traits because of the psychedelic use. Two single-item questions crafted by the research team, captured positive personality change and negative personality change, respectively, using a 5-point scale from 1 (not at all) to 5 (very much).

#### Relational change

Participants rated the extent to which their psychedelic experiences had influenced the perceived quality of their relationships with family, friends, themselves, society, and nature. Each domain was measured with a single item crafted by the research team for this study, rated on a 5-point Likert scale from 1 (not at all) to 5 (very much).

#### Psychedelic experience

To assess core experiential aspects, participants completed single-item ratings of mystical experience, personal meaningfulness, challenging experience, fearfulness, and perceived benefits from fearful experiences. All items were rated on a 5-point Likert-type scale (1 = not at all, 5 = extremely) and were adapted from well-established, validated measures commonly used in psychedelic research. To reduce respondent burden and survey fatigue, single items were selected to capture key experiential domains, yielding descriptive and exploratory data suitable for age-group comparisons. Participants also reported whether the psychedelic experience was among the top five most meaningful experiences of their lives, rated on a validated 7-point scale from 1 (like an everyday experience) to 7 (the single most meaningful experience).

### Data analysis

All analyses were conducted using Jamovi version 2.3.28.0. Given the heterogeneity within commonly used age categories during late adolescence and emerging adulthood, age was examined both categorically and continuously to explore whether observed patterns were sensitive to modelling approach [43, 44].

For primary analyses, participants were grouped as under 25 years and 25 years or older, reflecting prior developmental frameworks [7] and allowing comparison with existing literature (Izmi et al.,2024). In parallel, age was also included as a continuous variable in regression models to retain maximal information and to examine whether observed associations were robust to modelling approach. This strategy was adopted in light of the uneven age distribution within the younger group, particularly the small number of participants in mid-adolescence, which limited the feasibility of finer categorical subgroup analyses. All age-based analyses were conducted within an exploratory framework. Continuous age models were used to complement, rather than replace, categorical comparisons, and results were interpreted descriptively with attention to consistency across modelling approaches rather than formal hypothesis testing.

Descriptive statistics, independent samples t-tests, and ANCOVA were conducted to assess differences between age groups. Cohen’s d and partial eta squared (η²) were calculated as effect size measures. ANCOVA models controlled for relevant covariates identified in previous research such as personality and setting. Cohen’s d interpreted as small (< 0.15), medium (< 0.36), and large (> 0.65) (Lovakov, [45]). A correlation matrix was calculated with age as a continuous variable and effect-size interpretation small *r*>.12, medium *r* >.24, large *r* >.41 [46]. Linear regression was conducted with age as a continuous variable, adding Big Five as covariates. Assumptions checks using Normality tests and Q-Q plots in ANCOVA and linear regression reported in Supplements.

### Ethics

This study was approved by the Swedish Ethical Review Authority (Ref. No. 2024-01943-02). Participants were informed about the anonymous nature of the survey and their right to withdraw at any time. No identifying data were collected. Participants under 18 years of age provided informed consent without parental consent in accordance with national ethical guidelines for low-risk anonymous surveys involving adolescents.

## Results

Table 1 presents descriptive characteristic by age group adolescents (< 25) and adults (≥ 25) and Table 2 presents descriptive measures of well-being and Big Five traits and standardized mean differences (Cohen’s d). Adults reported higher harmony in life (small effect), whereas adolescents showed slightly higher neuroticism (small effect), and adults scored higher on agreeableness (approaching medium effect) and conscientiousness (small effect). Extraversion and openness showed negligible effects. Overall, the pattern indicates modest adult advantages in well-being and social/self-regulatory traits, alongside a small adolescent elevation in negative affectivity (see Table 2).

**Table 1:** Demographic characteristics

Variable	Age < 25		Age > 25	
	Mean	SD	Mean	SD
Age (years)	22.62	1.97	41.54	11.99
SES – upbringing (1–10)	5.65	2.01	5.71	1.98
SES – future expectation (1–10)	6.65	2.03	6.42	1.84
Sex/gender identity	***N***	***%***	***N***	***%***
Male	144	62.8%	789	58.3%
Female	80	34.9%	553	40.9%
Other identity	2	1.3%	3	0.2%

Participants were categorized into two age groups: adolescents (< 25 years; *N* = 134) and adults (≥ 25 years; *N* = 1,051). SES= Socioeconomic status rated on 1–10 scales. Percentages reflect within-age group proportions

**Table 2:** Comparison of adolescent (age < 25) and adult (age ≥ 25) psychedelic users in Well-being and Personality

Variables	Age < 25		Age > 25			
	M	SD	M	SD	Cohen’s d	*p*
The Harmony in Life Scale (1–7)	4.08	1.43	4.49	1.47	−0.28**	0.008
Life Satisfaction Index (1–5)	3.51	0.94	3.65	0.76	−0.13	0.207
Health Satisfaction (1–5)	3.53	1.02	3.64	0.96	−0.07	0.493
Neuroticism (1–5)	2.68	0.98	2.51	1.01	0.17*	0.016
Extraversion (1–5)	3.24	0.86	3.25	0.87	−0.01	0.892
Openness (1–5)	3.86	0.79	3.97	0.77	−0.14	0.053
Agreeableness (1–5)	3.81	0.68	4.06	0.67	−0.37***	< 0.001
Conscientiousness (1–5)	3.56	0.74	3.70	0.71	−0.21**	0.005

Values represent means (M), standard deviations (SD). P-value = probability of results being due to chance. *p <.05, **p <.01, ***p <.001. Cohen’s d = standardized mean difference, interpreted as small (> 0.15), medium (> 0.36), and large (> 0.65). <25 years; *N* = 134 and ≥ 25 years; *N* = 1051

Table 3 compares adolescents and adults self-reported effects related to psychedelic use. The Adverse Outcome Index was higher among adolescents (small-to-medium effect), and they endorsed depressed mood, sensory disturbances, persisting anxiety, confusion, sleeping problems, and feelings of unreality more frequently than adults (small effects to medium for unreality), all *p* ≤.006. Hyperactivation showed a small effect but was not statistically significant (*p* =.060). For personality change, groups did not differ in positive change, whereas adolescents reported more negative change than adults with a medium-to-large effect. In exploratory analyses of relationship changes, adults reported more improvement in family relationships (small effect). No between-group differences were observed for relationships with friends, self, society, or nature. Finally, in exploratory analysis of psychedelic experience and beneficial outcomes, there were no age differences in meaningfulness, challenging experiences, mystical experiences, self-change, or reporting a top life experience. Adolescents reported more fearful experiences and less perceived benefits from fearful experiences, each small in magnitude. Taken together, adolescents reported higher adverse-outcome and fear-related ratings, whereas groups were similar on meaningfulness, mystical experience, and related indices (see Table 3).

**Table 3:** Comparison of adolescent and adult psychedelic users on psychedelic use related effects

Variables	Age < 25			Age > 25					
	M	SD		M	SD	Cohen’s d		*p*	
Adverse Outcome Index	1.87	1.17		1.55	0.89	0.35***		0.001	
Feeling depressed	1.77	1.31		1.47	1.05	0.27**		0.006	
Sensory disturbances	1.81	1.27		1.51	1.06	0.27**		0.006	
Persisting anxiety	1.92	1.42		1.60	1.17	0.27**		0.006	
Confusion	1.89	1.35		1.51	1.08	0.33***		0.001	
Sleeping problems	1.89	1.49		1.50	1.10	0.34***		0.001	
Hyperactivation	1.75	1.27		1.55	1.10	0.18		0.060	
Feelings of unreality	2.15	1.45		1.70	1.15	0.37***		0.001	
Change to Personality									
Positive Change	3.17	1.26		3.33	1.34	−0.12		0.261	
Negative Change	2.01	1.10		1.51	0.95	0.52***		0.001	
Relationship Change									
To Family	3.41	1.10		3.64	0.97	−0.22**		0.008	
To Friends	3.73	1.10		3.75	0.97	−0.03		0.767	
To Yourself	4.07	1.06		4.10	1.04	−0.03		0.728	
To Society	3.28	1.10		3.44	1.01	−0.15		0.066	
To Nature	3.95	1.08		4.07	0.99	−0.12		0.153	
Psychedelic Experience									
Meaningfulness of Experience	3.88	1.24		3.95	1.25	−0.05		0.542	
Challenging Experience	2.99	1.34		2.97	1.40	0.02		0.835	
Mystical Experience	3.56	1.19		3.59	1.30	−0.02		0.844	
Self-Change from Experience	3.49	1.17		3.30	1.31	0.15		0.162	
Fearful Experience	2.49	1.26		2.28	1.27	0.17*		0.048	
Fear Experience with Benefits	3.73	1.35		3.45	1.47	0.20*		0.023	
Top Life Experience	5.59	1.65		5.55	1.75	0.02		0.767	

Values represent means (M), standard deviations (SD) for each age group. P-value = probability of results being due to chance. *p <.05, **p <.01, ***p <.001. Cohen’s d = standardized mean difference, interpreted as small (> 0.15), medium (> 0.36), and large (> 0.65). Adolescents (< 25 years; *N* = 134) and adults (≥ 25 years; *N* = 1051)

Table 4 summarizes an ANCOVA testing age group (< 25 vs. ≥25) with Big Five traits as covariates. The unadjusted age-group difference in Adverse Outcome Index was significant; after adjusting for personality, the age effect remained significant but attenuated, indicating unique age-related variance beyond traits. Among the covariates, neuroticism accounted for the largest unique share of variance, with agreeableness and openness also contributing; conscientiousness and extraversion were not significant. Assumption checks (see Supplements) indicated non-normal residuals; given the large sample and ANCOVA’s robustness to mild non-normality, we proceeded and emphasize effect-size interpretation.

**Table 4:** ANCOVA of adverse outcomes following psychedelic use

Predictor variable	F	*p*	η²
Age Group (unadjusted)	12.41	< 0.001	0.01
Age Group (adjusted)	5.82	< 0.01	0.01
Big Five Neuroticism	22.23	< 0.001	0.03
Big Five Extraversion	0.11	0.74	0.00
Big Five Openness	11.89	< 0.001	0.01
Big Five Agreeableness	17.44	< 0.001	0.02
Big Five Conscientiousness	2.97	0.08	0.00

F = Fischer’s ratio between-group versus within-group variance. P-value = significance test by ANCOVA. η² = partial eta squared, proportion of variance explained in the dependent variable. *N* = 1185

Table 5 presents bivariate pearson correlations with age (years) treated as a continuous variable. Across outcomes, the associations with age and adverse outcomes were uniformly negligible when interpreted against empirically derived benchmarks for social/personality research [45]. However, age showed small effects in well-being and personality trait neuroticism, but no significant relations with adverse outcomes, except a small effect on perceived negative personality change. Correlations with experience characteristics found a small effect on fearful experience with related benefits, but otherwise negligible on meaningfulness, mystical/challenging experiences, self-change, “top life” experience. For changes to relations with family, friends, self, society, or nature, no effects were shown. No age correlation approached the medium range (*r* ≥.24). Given the large sample, very small effects May attain statistical significance; interpretation therefore prioritizes effect size and pattern over p values (see table 5) 

**Table 5:** Correlation Matrix for Age (Continuous) and Study Outcomes

	1	2	3	4	5	6	7	8	9	10	11	12	13	14	15	16	17	18	19	20	21	22	23	24	25	26	27	28	29
1. Age	—																												
2. HILS	*0.15*	*—*																											
3. Neuroticism	*− 0.12*	*− 0.51*	*− 0.48*	*—*																									
4. Extraversion	*− 0.04*	*0.39*	*0.48*	*− 0.39*	*—*																								
5. Openness	*0.09*	*0.10*	*0.20*	*− 0.12*	*0.20*	*—*																							
6. Agreeableness	*0.11*	*0.10*	*0.17*	*− 0.14*	*0.29*	*0.45*	*—*																						
7. Conscientiousness	*0.09*	*0.33*	*0.33*	*− 0.33*	*0.35*	*0.20*	*0.30*	*—*																					
8. Adverse outcome index	*0.00*	*− 0.06*	*− 0.09*	*0.21*	*− 0.12*	*− 0.24*	*− 0.25*	*− 0.09*	*—*																				
9. Feeling depressed	*0.00*	*− 0.10*	*− 0.13*	*0.24*	*− 0.14*	*− 0.23*	*− 0.25*	*− 0.11*	*0.81*	*—*																			
10. Sensory disturbances	*0.01*	*0.00*	*0.01*	*0.08*	*− 0.04*	*− 0.14*	*− 0.20*	*− 0.07*	*0.78*	*0.58*	*—*																		
11. Persisting anxiety	*0.02*	*− 0.05*	*− 0.09*	*0.25*	*− 0.10*	*− 0.26*	*− 0.22*	*− 0.09*	*0.86*	*0.73*	*0.57*	*—*																	
12. Confusion	*− 0.01*	*− 0.04*	*− 0.07*	*0.14*	*− 0.09*	*− 0.20*	*− 0.21*	*− 0.08*	*0.85*	*0.61*	*0.59*	*0.71*	*—*																
13. Sleeping problems	*− 0.02*	*− 0.05*	*− 0.06*	*0.20*	*− 0.13*	*− 0.24*	*− 0.22*	*− 0.09*	*0.83*	*0.62*	*0.55*	*0.70*	*0.64*	*—*															
14. Hyperactivation	*0.04*	*− 0.03*	*− 0.03*	*0.12*	*− 0.06*	*− 0.14*	*− 0.20*	*− 0.03*	*0.78*	*0.53*	*0.56*	*0.57*	*0.59*	*0.65*	*—*														
15. Feelings of unreality	*− 0.03*	*− 0.05*	*− 0.07*	*0.17*	*− 0.09*	*− 0.12*	*− 0.17*	*− 0.04*	*0.83*	*0.58*	*0.60*	*0.63*	*0.71*	*0.58*	*0.61*	*—*													
16. Positive personality change	*− 0.04*	*0.16*	*0.19*	*− 0.05*	*0.15*	*0.31*	*0.17*	*0.10*	*0.00*	*− 0.03*	*0.05*	*− 0.08*	*− 0.02*	*− 0.03*	*0.05*	*0.05*	*—*												
17. Negative personality change	*− 0.13*	*− 0.04*	*− 0.12*	*0.22*	*− 0.03*	*− 0.33*	*− 0.32*	*− 0.16*	*0.38*	*0.33*	*0.30*	*0.35*	*0.30*	*0.30*	*0.24*	*0.26*	*− 0.05*	*—*											
18. Relationship change – family	*− 0.05*	*0.16*	*0.23*	*− 0.10*	*0.18*	*0.21*	*0.15*	*0.05*	*− 0.19*	*− 0.22*	*− 0.06*	*− 0.21*	*− 0.14*	*− 0.15*	*− 0.13*	*− 0.13*	*0.42*	*− 0.12*	*—*										
19. Relationship change – friends	*− 0.11*	*0.07*	*0.13*	*− 0.07*	*0.20*	*0.26*	*0.18*	*0.05*	*− 0.20*	*− 0.21*	*− 0.10*	*− 0.24*	*− 0.18*	*− 0.16*	*− 0.09*	*− 0.13*	*0.39*	*− 0.12*	*0.68*	*—*									
20. Relationship change – yourself	*− 0.11*	*0.10*	*0.16*	*− 0.09*	*0.13*	*0.34*	*0.17*	*0.08*	*− 0.26*	*− 0.28*	*− 0.11*	*− 0.31*	*− 0.22*	*− 0.24*	*− 0.13*	*− 0.18*	*0.51*	*− 0.22*	*0.61*	*0.62*	*—*								
21. Relationship change – society	*0.02*	*0.12*	*0.14*	*− 0.09*	*0.16*	*0.20*	*0.12*	*0.11*	*− 0.14*	*− 0.16*	*− 0.04*	*− 0.15*	*− 0.15*	*− 0.14*	*− 0.06*	*− 0.09*	*0.31*	*− 0.09*	*0.53*	*0.52*	*0.51*	*—*							
22. Relationship change – nature	*− 0.09*	*0.10*	*0.19*	*− 0.08*	*0.16*	*0.39*	*0.25*	*0.09*	*− 0.23*	*− 0.25*	*− 0.10*	*− 0.27*	*− 0.22*	*− 0.22*	*− 0.11*	*− 0.12*	*0.49*	*− 0.19*	*0.58*	*0.60*	*0.70*	*0.48*	*—*						
23. Meaningfulness of experience	*− 0.14*	*0.07*	*0.14*	*− 0.10*	*0.23*	*0.35*	*0.25*	*0.08*	*− 0.23*	*− 0.21*	*− 0.12*	*− 0.26*	*− 0.21*	*− 0.22*	*− 0.14*	*− 0.15*	*0.56*	*− 0.13*	*0.44*	*0.41*	*0.58*	*0.30*	*0.50*	*—*					
24. Challenging experience	*− 0.05*	*0.03*	*0.03*	*0.02*	*0.12*	*0.10*	*0.03*	*0.06*	*0.11*	*0.08*	*0.11*	*0.11*	*0.13*	*0.04*	*0.06*	*0.14*	*0.14*	*0.15*	*0.11*	*0.10*	*0.10*	*0.04*	*0.10*	*0.22*	*—*				
25. Mystical experience	*− 0.06*	*0.13*	*0.18*	*− 0.09*	*0.21*	*0.22*	*0.13*	*0.12*	*− 0.02*	*− 0.04*	*0.04*	*− 0.09*	*− 0.02*	*− 0.06*	*0.03*	*0.03*	*0.46*	*− 0.02*	*0.27*	*0.25*	*0.33*	*0.20*	*0.36*	*0.44*	*0.17*	*—*			
26. Self-change from experience	*− 0.10*	*0.13*	*0.19*	*− 0.02*	*0.15*	*0.26*	*0.12*	*0.08*	*0.06*	*0.00*	*0.12*	*− 0.04*	*0.05*	*− 0.01*	*0.08*	*0.12*	*0.64*	*0.02*	*0.35*	*0.30*	*0.48*	*0.27*	*0.45*	*0.59*	*0.20*	*0.57*	*—*		
27. Fearful experience	*0.00*	*0.03*	*0.00*	*0.14*	*− 0.04*	*− 0.14*	*− 0.14*	*− 0.07*	*0.34*	*0.28*	*0.26*	*0.35*	*0.29*	*0.26*	*0.15*	*0.32*	*0.05*	*0.25*	*− 0.11*	*− 0.15*	*− 0.18*	*− 0.09*	*− 0.15*	*− 0.13*	*0.45*	*0.02*	*0.08*	*—*	
28. Fearful experience with benefits	*− 0.21*	*0.10*	*0.13*	*− 0.05*	*0.20*	*0.24*	*0.19*	*0.07*	*− 0.11*	*− 0.12*	*− 0.04*	*− 0.14*	*− 0.08*	*− 0.14*	*− 0.05*	*− 0.04*	*0.54*	*− 0.03*	*0.35*	*0.32*	*0.43*	*0.23*	*0.36*	*0.53*	*0.37*	*0.36*	*0.54*	*0.17*	*—*
30. Top life experience	*− 0.10*	*0.05*	*0.11*	*− 0.13*	*0.16*	*0.31*	*0.27*	*0.07*	*− 0.20*	*− 0.22*	*− 0.16*	*− 0.21*	*− 0.13*	*− 0.19*	*− 0.15*	*− 0.11*	*0.38*	*− 0.17*	*0.32*	*0.31*	*0.40*	*0.21*	*0.38*	*0.51*	*0.21*	*0.35*	*0.41*	*− 0.04*	*0.40*

Pearson correlations among study variables with age (years) modelled as a continuous variable. Effect-size interpretation follows small (|r| ≥ 0.12), medium (|r| ≥ 0.24), large (|r| ≥ 0.41)

Table 6 summarizes the multiple regression relating age and Big Five traits to adverse outcomes. The regression model accounted for 11% of the variance in adverse outcomes. By conventional benchmarks this represents a small-to-moderate effect [46], indicating that while age and personality contribute meaningfully to the prediction of adverse outcomes, the majority of variance remains unexplained. In terms of predictors, higher neuroticism was associated with more adverse outcomes, whereas higher agreeableness and higher openness were associated with fewer adverse outcomes. Age, extraversion, and conscientiousness did not contribute uniquely.

**Table 6:** Linear regression predicting adverse outcomes following psychedelic use

Model fit		*R*	*R*²	Adjusted *R*²	
		0.32	0.11	0.10	
Predictor	***B***	***SE***	***t***	***p***	***β***
Intercept	2.49	0.31	8.14	< 0.001	
Age	0.01	0.01	0.71	0.48	0.03
Big Five Neuroticism	0.17	0.03	4.91	< 0.001	0.19
Big Five Extraversion	0.02	0.04	0.60	0.55	0.02
Big Five Openness	−0.17	0.05	−3.40	< 0.001	−0.14
Big Five Agreeableness	−0.24	0.06	−4.47	< 0.001	−0.18
Big Five Conscientiousness	0.07	0.05	1.43	0.15	0.06

Dependent variable = Adverse Outcome Index. Age (continuous years). Model fit: R = multiple correlation between observed and predicted values; R² = proportion of variance explained; adjusted R² = R² corrected for model complexity. B = unstandardized coefficient, SE = standard error of the B; t tests whether B = 0 (two-tailed). p values are reported exactly where possible (values < 0.001 shown as *p* <.001). Standardized coefficients (β) are reported

## Discussion

This study examined differences in adverse and beneficial outcomes of psychedelic use between adolescents and adults, with a particular focus on the role of personality traits. Several key findings emerged in relation to the research objectives. First, adolescents reported more adverse outcomes (see Table 3), compared to adults (d = 0.35) which align with previous publications [2, 3]. A novel finding of medium effect size was increased perceived negative personality change related to psychedelic use in adolescents (d = 0.52) and higher report of fearful experiences (d = 0.17*)*. Fearful, or challenging experiences have been associated with potential for more beneficial outcomes [47], although in this study the adolescents reported less benefit of fearful experiences than adults (d = 0.20). In summary, the results continue to indicate that psychedelic use among adolescents may come with an increased risk of adverse outcomes in comparison to adults.

Second, positive outcomes such as meaningfulness and improvements in relationships with friends, self, or nature were comparable across groups, suggesting that adolescents share the potential for benefit related to psychedelic experience with adults. Qualitative work with adolescents with psychedelic experiences (Sjostrom et al., in preparation) also indicates that individual adolescents report lasting personal benefits, including improved self-understanding and relational functioning. In this light, it is worth noting that adolescent use of psychedelics is not inherently only a risk for harm, but also a window for personal development and well-being in companion with a need for age-informed harm reduction strategies. Third, the ANCOVA indicated that age group remained a significant predictor on adverse outcomes after adjustment for personality traits (see Table 4) that are known correlates of psychedelic-use–related outcomes. However, personality—particularly higher neuroticism—accounted for a larger unique share of variance than age (η² ≈ 0.03 for neuroticism vs. ≈ 0.01 for age group; roughly threefold). Effects for agreeableness (η² ≈ 0.02) and openness (η² ≈ 0.01) were also small, and extraversion and conscientiousness were negligible. Thus, all ANCOVA effects were small by conventional benchmarks. This pattern aligns with prior work showing neuroticism as a consistent correlate of challenging psychedelic experiences and adverse outcomes [37, 38] and explaining additional variance in mental-health outcomes among psychedelic users [15, 40]. Because partial η² values are not additive, they do not yield a single “total variance explained.” Converging evidence from the companion linear regression (Table 6) showed modest overall explanatory power (R² ≈ 0.11), underscoring that age and personality capture only a limited portion of the variability in adverse outcomes. Other factors likely contribute, including psychiatric vulnerability, frequency and intensity of psychedelic use, set and setting, and the availability of integration support. Incorporating such covariates in future research could provide a more comprehensive model of individual risk. Importantly, the persistence of an age effect after adjustment for personality indicates that developmental stage (adolescence) may contribute incrementally to risk, theoretically related to emotional reactivity and later maturation of regulatory systems during adolescence [10], which could magnify the psychological challenges of psychedelic experiences and risk for adverse outcomes. In addition, adolescents typically show higher mean levels of neuroticism (and other trait differences) relative to adults [39, 48] associated with adverse outcomes. Disentangling these pathways will require longitudinal and mechanistic designs including significant co-variates to further evaluate adolescence as a risk factor for adverse outcomes in relation to psychedelic use.

An exploratory finding of the present study was that participants under 25 years of age reported fewer improvements in family relationships following psychedelic use compared to adults (d = 0.22), whereas no age-group differences were observed for relationships with friends, self, society, or nature. Although relational outcomes were not a primary focus of the study, this pattern warrants careful interpretation within a developmental and contextual framework. Adolescence are developmental periods marked by ongoing renegotiation of autonomy, boundaries, and identity within the family system. Extensive developmental research indicates that these processes are commonly accompanied by increased parent–child conflict, heightened individuation, and reduced disclosure, even in otherwise supportive families [49, 50]. Within such contexts, intrapersonal psychological changes—such as increased emotional insight, self-reflection, or shifts in self-concept—may not readily translate into observable improvements in family relationships. From a psychedelic research perspective, this interpretation is further supported by work emphasizing the central role of set and setting in shaping outcomes [51]. Psychedelic experiences are deeply embedded within social, cultural, and relational contexts, and their interpersonal effects may depend on factors such as openness, disclosure, and relational safety. For adolescents, psychedelic use may occur in contexts characterized by secrecy, stigma, or limited parental involvement, potentially constraining the expression or integration of intrapersonal insights within family relationships. In such cases, benefits may initially manifest intrapersonally rather than interpersonally or emerge in relational domains outside the family (e.g., friendships or relationship to self).

Last, results on “age effects” were sensitive to how age was operationalized. A theory-driven dichotomous comparison (< 25 vs. ≥25) yielded a small effect even after adjustment; in contrast, continuous modelling indicated negligible bivariate correlations with age (see Table 5) and no linear age effect once personality was analysed as a predictor in a regression model (see Table 6). This divergence underscores a familiar trade-off. On the one hand, a < 25/≥25 cutoff is conceptually motivated, mapping onto contemporary developmental research [7, 8]. On the other hand, categorizing a continuous predictor discards more variance in the data and might produce different results [43, 44]. Our results—small to moderate effects when comparing adolescents with adults as age groups, but negligible effects when using age as a continuous variable —illustrate why both perspectives can be important when exploring age-related effects by psychedelics.

Taken together these results underscore the value of age and trait focus on relation to psychedelic use in adolescence. Future research should (a) test mechanistic pathways using longitudinal and, where feasible, neuroimaging designs (b) incorporate richer covariates—including prior psychopathology, trauma exposure, substance type, dose/frequency, set/setting, family system perspectives and integration practices—and (c) model nonlinearity in age alongside personality to capture potential thresholds effects. Taken together, the evidence points to a growing public-health need for evidence-based, developmentally informed supportive frameworks for adolescents who engage with psychedelics.

## Limitations

The present findings should be interpreted considering several important limitations that directly shape the conclusions that can be drawn from this study. First, the cross-sectional and observational nature of the data precludes causal inference. Although age-group differences and associations with personality traits were observed, these patterns cannot be interpreted as evidence that age or personality causes specific psychedelic-related outcomes. Rather, they reflect descriptive associations within a self-selected sample at a single point in time.

Second, the age distribution of the sample imposes clear constraints on developmental interpretation. While participants were categorized as under 25 versus 25 and older, the younger group was heavily weighted toward late adolescents/emerging adults aged 20–24, with very few participants aged 16–17. As a result, findings attributed to the < 25 group should not be interpreted as representative of mid-adolescence, but rather as reflecting late adolescence and emerging adulthood. This limitation reduces the precision with which developmental stage differences can be inferred and underscores the need for targeted sampling in future research.

Third, the study relied on a convenience sample recruited online, which introduces the possibility of self-selection bias. Participants who choose to complete surveys about psychedelic use may differ systematically from non-participants in terms of personality, attitudes toward psychedelics, or experiences with adverse outcomes. Consequently, the prevalence and patterns of outcomes reported here should not be generalized to broader populations of adolescents or adults.

Fourth, several relevant covariates were not assessed, including substance type, dose, frequency of use, psychiatric history, integration practices, and age at first use. The absence of these variables limits the ability to contextualize reported outcomes and may confound observed associations. For example, age-related differences may partially reflect differences in substance exposure or usage patterns that could not be accounted for in the present analyses.

Fifth, all measures were based on retrospective self-report on personal psychedelic experiences, which may be affected by significant recall bias, social desirability, and differential interpretation of survey items across age groups.

Sixth, it is important to note that relational outcomes in this study were assessed using single-item self-report measures crafted by the research team and were explicitly framed as exploratory in this study. Consequently, these findings should be interpreted cautiously and regarded as hypothesis-generating rather than definitive.

Seventh, although we implemented basic bot/inattention safeguards (such as CAPTCHA and response screening), residual uncertainty remains inherent in anonymous online self-report samples.

Finally, although personality traits were included as covariates, these were used in an exploratory manner to assess explanatory patterns rather than as comprehensive controls. While personality accounted for substantial variance in outcomes, this does not imply that personality exhaustively explains age-related differences, nor that unmeasured factors are unimportant.

## Conclusion

Within several important limitations, the present exploratory study demonstrated that adolescents as a group reported more frequent adverse outcomes following psychedelic use than adults, whereas positive outcomes appeared broadly comparable. Individual differences, particularly higher neuroticism, explained differences in adverse outcomes more than the age group alone. Notably, the age group effect on adverse outcomes attenuated when age was modelled as a continuous variable. The results argue for future longitudinal and controlled research, meanwhile encourage age-sensitive, yet trait-focused, harm reduction and public health discourse in relation to increasing use of classical psychedelics in adolescent populations.

## Supplementary Information

Supplementary Material 1.

## Data Availability

No datasets were generated or analysed during the current study.
