# Supplementary material for: Adverse outcomes following psychedelic use in adolescents and adults: associations with age and personality traits
Source: Child Adolesc Psychiatry Ment Health. 2026 Feb 24;20:32. doi: 10.1186/s13034-026-01048-x (PMC12958705; doi:10.1186/s13034-026-01048-x)
Supplement: Supplementary file 1 — Supplementary Material 1. [file 13034_2026_1048_MOESM1_ESM.docx]

**Supplement Materials**

**Assumption Checks ANCOVA and Linear Regression Models**

| Normality Test (Shapiro-Wilk) | |
| --- | --- |
| Statistic | **p** |
| *0.98* | *<.001* |

**Q-Q Plot**


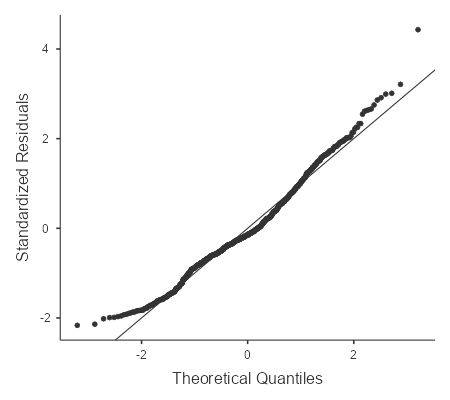


# **Country of Origin Frequencies**

| **Country** | **Count** |
| --- | --- |
| United States of America | 655 |
| Sweden | 440 |
| Finland | 22 |
| United Kingdom | 10 |
| Denmark | 10 |
| Canada | 9 |
| Netherlands | 8 |
| Portugal | 6 |
| Switzerland | 6 |
| Spain | 3 |
| South Africa | 3 |
| India | 2 |
| Thailand | 2 |
| Germany | 2 |
| Barbados | 1 |
| Malta | 1 |
| France | 1 |
| Russia | 1 |
| Norway | 1 |
| Chile | 1 |
| Bulgaria | 1 |
| Mexico | 1 |
| Scandinavia (non-country) | 1 |

*Note.* Counts represent the number of respondents reporting each country of origin. United States entries include all variations of naming (e.g., USA, U.S.A., America). Scandinavia is listed as reported and does not represent a single sovereign country.

**Survey items assessing psychedelic experience characteristics and outcomes, listed in Table 3.**

| Survey item |
| --- |
| Did you experience any side effects in the days/weeks after your psychedelic experience in the body and/or mind? [Depression] |
| Did you experience any side effects in the days/weeks after your psychedelic experience in the body and/or mind? [Persistent changes in vision/hearing/touch] |
| Did you experience any side effects in the days/weeks after your psychedelic experience in the body and/or mind? [Worry/anxiety] |
| Did you experience any side effects in the days/weeks after your psychedelic experience in the body and/or mind? [Confusion] |
| Did you experience any side effects in the days/weeks after your psychedelic experience in the body and/or mind? [Sleep problems] |
| Did you experience any side effects in the days/weeks after your psychedelic experience in the body and/or mind? [Revved up] |
| Did you experience any side effects in the days/weeks after your psychedelic experience in the body and/or mind? [Feeling of unreality] |
| How has this psychedelic experience affected the quality of your relationship with family? |
| How has this psychedelic experience affected the quality of your relationship with friends? |
| How has this psychedelic experience affected the quality of your relationship to yourself? |
| How has this psychedelic experience affected the quality of your relationship with society? |
| How has this psychedelic experience affected the quality of your relationship with nature? |
| To what extent do you experience that your personality has changed for the better? |
| To what extent do you experience that your personality has changed for the worse? |
| How personally meaningful was the psychedelic experience that affected you the most? |
| How personally challenging was the psychedelic experience that affected you the most? |
| To what extent would you describe the experience as mystical? |
| To what extent would you describe that your experience of the self changed? |
| To what extent would you describe this experience as fearful? |
| To what extent would you perceive the fearful parts of the experience to have brought anything good? |
